# Reduced expression of BMP3 contributes to the development of pulmonary fibrosis and predicts the unfavorable prognosis in IIP patients

**DOI:** 10.18632/oncotarget.20083

**Published:** 2017-08-09

**Authors:** Xiaoting Yu, Pan Gu, Ziling Huang, Xia Fang, Ying Jiang, Qun Luo, Xia Li, Xuyou Zhu, Mengna Zhan, Junbang Wang, Lichao Fan, Rongchang Chen, Juehua Yu, Yingying Gu, Aibin Liang, Xianghua Yi

**Affiliations:** ^1^ Department of Pathology, Tongji Hospital, Tongji University School of Medicine, Shanghai 200065, China; ^2^ Department of Biotherapy, Tongji Hosptial, Tongji University School of Medicine, Shanghai 200065, China; ^3^ The State Key Laboratory of Respiratory Disease, Guangzhou Institute of Respiratory Disease, the First Affiliated Hospital, Guangzhou Medical University, Guangzhou, Guangdong 510120, China; ^4^ Department of Respiratory, Shanghai Pulmonary Hospital, Tongji Universiy School of Medicine, Shanghai 200433, China; ^5^ Department of Pathology, Zhongshan Hospital, Fudan University, Shanghai 200032, China; ^6^ Stem Cell Translational Research Center, Tongji Hospital, Tongji University School of Medicine, Shanghai 200065, China; ^7^ Department of Respiratory, Tongji Hospital, Tongji University School of Medicine, Shanghai 200065, China

**Keywords:** bone morphogenetic protein 3/BMP3, transforming growth factor-β/TGF-β, idiopathic pulmonary fibrosis/IPF, idiopathic nonspecific interstitial pneumonia/INSIP

## Abstract

Idiopathic pulmonary fibrosis (IPF) and idiopathic nonspecific interstitial pneumonia (INSIP) are two related diseases involving varying degrees of pulmonary fibrosis with no effective cure. Bone morphogenetic protein 3 (BMP3) is a member of the transforming growth factor-β (TGF-β) super-family, which has not been implicated in pulmonary fibrosis previously. In this study, we aimed to investigate the potential role of BMP3 playing in pulmonary fibrosis from clinical diagnosis to molecular signaling regulation. RNA sequencing was performed to explore the potential biomarker of IIP patients. The expression of BMP3 was evaluated in 83 cases of IPF and INSIP by immunohistochemistry. The function of BMP3 was investigated in both fibroblast cells and a bleomycin-induced murine pulmonary fibrosis model. The clinical relevance of BMP3 expression were analyzed in 47 IIP patients, which were included in 83 cases and possess more than five-year follow-up data. Both RNA-sequencing and immunohistochemistry staining revealed that BMP3 was significantly down-regulated in lung tissues of patients with IPF and INSIP. Consistently, lower expression of BMP3 also was found in pulmonary fibrotic tissues of bleomycin-induced mice model. Up-regulation of BMP3 prevented pulmonary fibrosis processing through inhibiting cellular proliferation of fibroblasts as well as TGF-β1 signal transduction. Finally, the relatively higher expression of BMP3 in IPF patients was associated with less/worse mortality. Intravenous injection of recombinant BMP3. Taken together, our results suggested that the low expression level of BMP3 may indicate the unfavorable prognosis of IPF patients, targeting BMP3 may represent a novel potential therapeutic method for pulmonary fibrosis management.

## INTRODUCTION

Idiopathic interstitial pneumonias (IIPs) are a group of interstitial lung diseases of unknown etiology that are characterized by varying degrees of chronic inflammation and progressive fibrosis of lung parenchyma [1, 2]. Idiopathic pulmonary fibrosis (IPF) and idiopathic nonspecific interstitial pneumonia (INSIP) are two major sub-types of IIPs [3, 4]. IPF is histopathologically defined by the presence of the typical form of pulmonary fibrosis and often results in death within 3–5 years of diagnosis [1]. INSIP is universally associated with a more cellular interstitial pneumonia with or without accompanying fibrosis and occurs earlier in life with a better prognosis than IPF [5–7]. Although some patients have a certain response to corticosteroid the effect is limited. Therefore, it has been suggested that efforts to combating IPF and INSIP should be aimed at exploring potential anti-fibrotic treatment strategies [8, 9].

A number of cytokines, including interleukins (ILs), transforming growth factor-β (TGF-β), and chemokines [10–14], secreted by lung epithelial cells, endothelial cells, stromal cells, and many types of activated inflammatory cells are known to be involved in the progress of pneumonia-related inflammation and pulmonary fibrosis. It is well known that TGF-β not only play important roles in regulating several physiological processes of the lung development, but also associated with a variety of pulmonary diseases, including fibrosis [15, 16]. Interestingly, increased evidences shown that Bone morphogenetic proteins (BMPs), as the members of TGF-β superfamily, are endogenous antagonism of TGF-β signaling. Physiologically, they are required for the maintenance of tissue homeostasis and regeneration after injury. [17]. Besides, they also heavily involved in the development of bone, cartilage, lung, and other organs in rodents [18]. Accumulating evidence suggests that BMPs participate in the processes of a variety of organ fibroses [19, 20]. BMP2 significantly attenuates TGF-β–induced renal fibrosis in rodents by modulating epithelial-mesenchymal transition (EMT) [21]. BMP6 was recently defined as a key regulator of renal fibrosis [22]. Augmenting the expression of BMP7 can reverse chronic renal injury via inhibition of TGF-β–mediated EMT [23]. In renal fibrosis mouse models, BMP7 was shown to suppress the fibrotic process by inhibiting the TGF-β/Smad signaling pathway, which plays an essential role in converting fibroblasts into large numbers of myofibroblasts leading to fibrosis [24–28]. Although these studies have uncovered important roles of BMPs in organ fibroses, including pulmonary fibrosis in rodent models, the clinical relevance of BMPs in pulmonary fibrosis diseases, including IPF and INSIP, were under investigation.

In the present study, RNA sequencing of lung tissue samples from IPF and INSIP patients was performed and the transcriptome of fibrosis lungs was compared to that of normal lungs. The expression of BMP3, which has not been previously reported to regulate fibrosis, was further evaluated in fibrotic lungs in 83 patients including 46 cases of IPF and 37 cases of INSIP by immunohistochemistry. The role of BMP3 in the pathogenesis of pulmonary fibrosis *in vivo* was determined using a bleomycin–induced murine pulmonary fibrosis model [29] The bleomycin animal model is widely used in the assessment of potential antifibrotic agents. A large number of compounds have been shown to prevent fibrotic progression in this model and have been suggested to qualify for clinical use. And we used primary mice fibroblasts culture to find the role of BMP3 *in vitro*. Finally, the clinical relevance of BMP3 was analyzed in 47 patients, which were cohort of 83 cases and have integrated five years of follow-up data.

## RESULTS

### Identification of genes dysregulated in lung tissues from IPF and INSIP patients

The illumina mRNA sequencing approach was used to determine the relative abundance of various genes in IIPs. For each tissue sample, 8.3 ± 1.0 million reads with an average read length of around 50 bp was generated to ensure sufficient and saturating sequencing depth. The reads were aligned with the human reference genome (GrCH37, Ensemble build 74) using Tophat version 2.0.12, yielding an average mapping rate of 94.7 ± 2.5%. Gene expression levels, which were represented as fragments per kilo-base per million mapped reads (FPKM), were obtained for 63, 783 genes/transcripts annotated using the Ensemble GrCH37 database release 74. The global gene expression profiles indicated that the gene expression patterns in diseased lung samples were distinct compared with those of healthy lung samples (Supplementary Figure 1A). Using a cut-off *P*-value < 0.01 and fold-change > 1.5, a total of 1652 differentially expressed genes were identified by comparing healthy and diseased lung tissue samples. Overall, 671 genes were up-regulated and 981 were down-regulated in fibrotic lungs (GSE73189). Besides, when 5156 differentially expressed genes were subjected to supervised weighted gene co-expression network analyses (WGCNA) based on FDR < 0.05 [34], four gene clusters (modules) were identified (Supplementary Figure 1B–1C), the cyan module was highly correlated with and expressed in normal lung tissues, whereas the brown module was highly correlated with lungs affected by IIPs.

**Figure 1:**
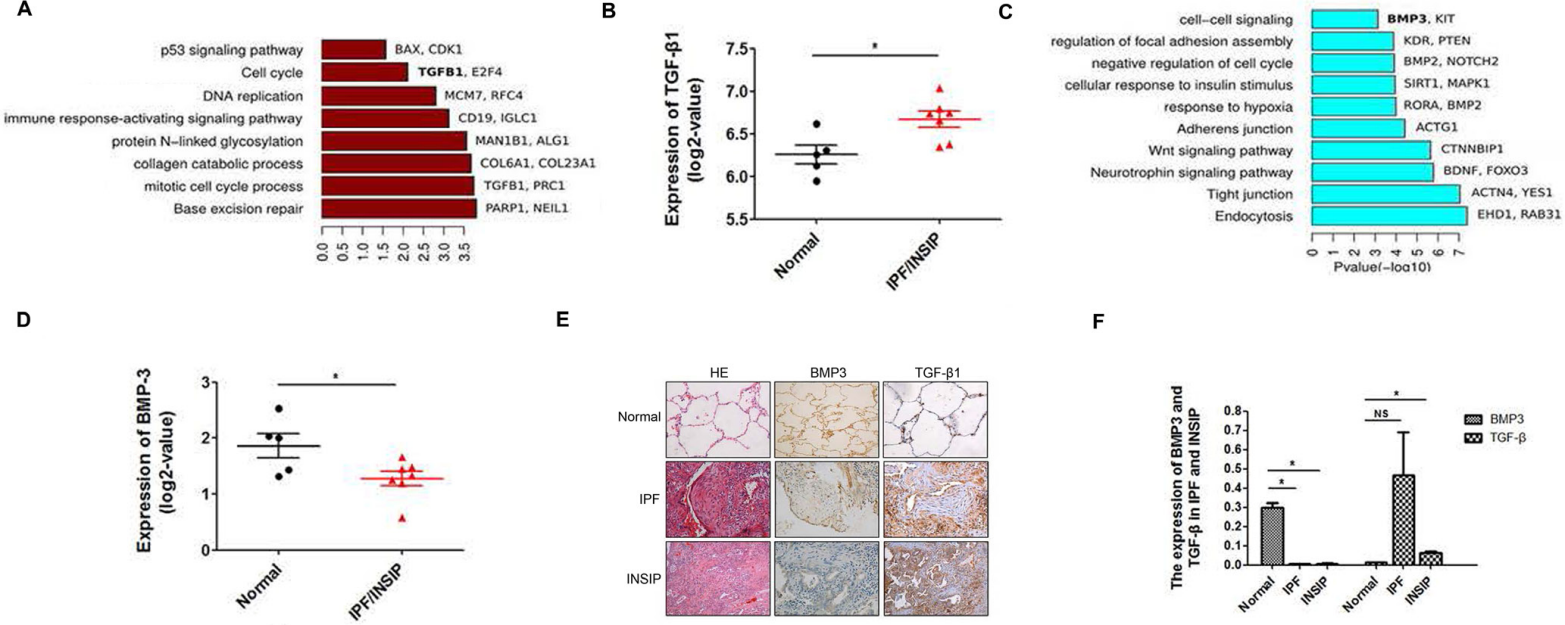
Bioinformatics analysis based on RNA-seq data of IPF and INSIP lung specimens (**A**) Gene ontology analysis of dark red modules, the bar length indicates *P*-value (Fisher's Exact Test, -log10 transferred). (**B**) The log2-FC values of *TGF-β*1 expression in the normal healthy and IIP patient groups. (5 normal healthy and 7 IIP patients) (**C**) Gene ontology analysis of cyan modules, the bar length indicates *P*-value (Fisher's Exact Test, -log10 transferred). (**D**) The log2-FC values of *BMP3* expression in the normal and IIP patient groups. (5 normal healthy and 7 IIP patients). (**E–F**) BMP3 and TGF-β1 expression in patients with IPF and INSIP as detected using IHC (IPF *n* = 46, INSIP *n* = 37), with at least five fields randomly selected and quantified by two pathologists under blinded conditions (*p < 0.05).

Gene ontology analysis revealed that the brown module, which was upregulated in lungs of IIP patients, was enriched for genes involved in DNA repair, mitotic cell cycle process, DNA replication, p53 signaling pathways, and others (Figure 1A). The well-known pro-fibrotic factor *TGF-β1* [15, 19] was detected in the brown module and likely caused increased proliferation of lung fibroblasts, thereby leading to pulmonary fibrosis (Figure 1A and B, P = 0.036). On the other hand, the cyan module including normal lung-specific genes was enriched for genes related to tight junctions, neurotrophin signaling, adherens junctions, negative regulation of cell cycle, and cell–cell signaling. In this module, in addition to the well-known anti-fibrotic factor *BMP2*, the related gene *BMP3* was present and was significantly downregulated in IIP lungs with a *p* value < 0.05 (Figure 1C and 1D, P = 0.015). Next, RNA-seq data was further validated in 83 IIP patient tissues by immunohistochemistry technique. The result revealed that BMP3 expression was down-regulated in both IPF and INSIP, whereas TGF-β expression was up-regulated (Figure 1E and 1F). Taken together, these data indicate that abnormal expression of BMP3 may play a critical role in the pathogenesis of IIPs.

### BMP3 was decreased in a bleomycin-induced murine pulmonary fibrosis model

**Figure 2:**
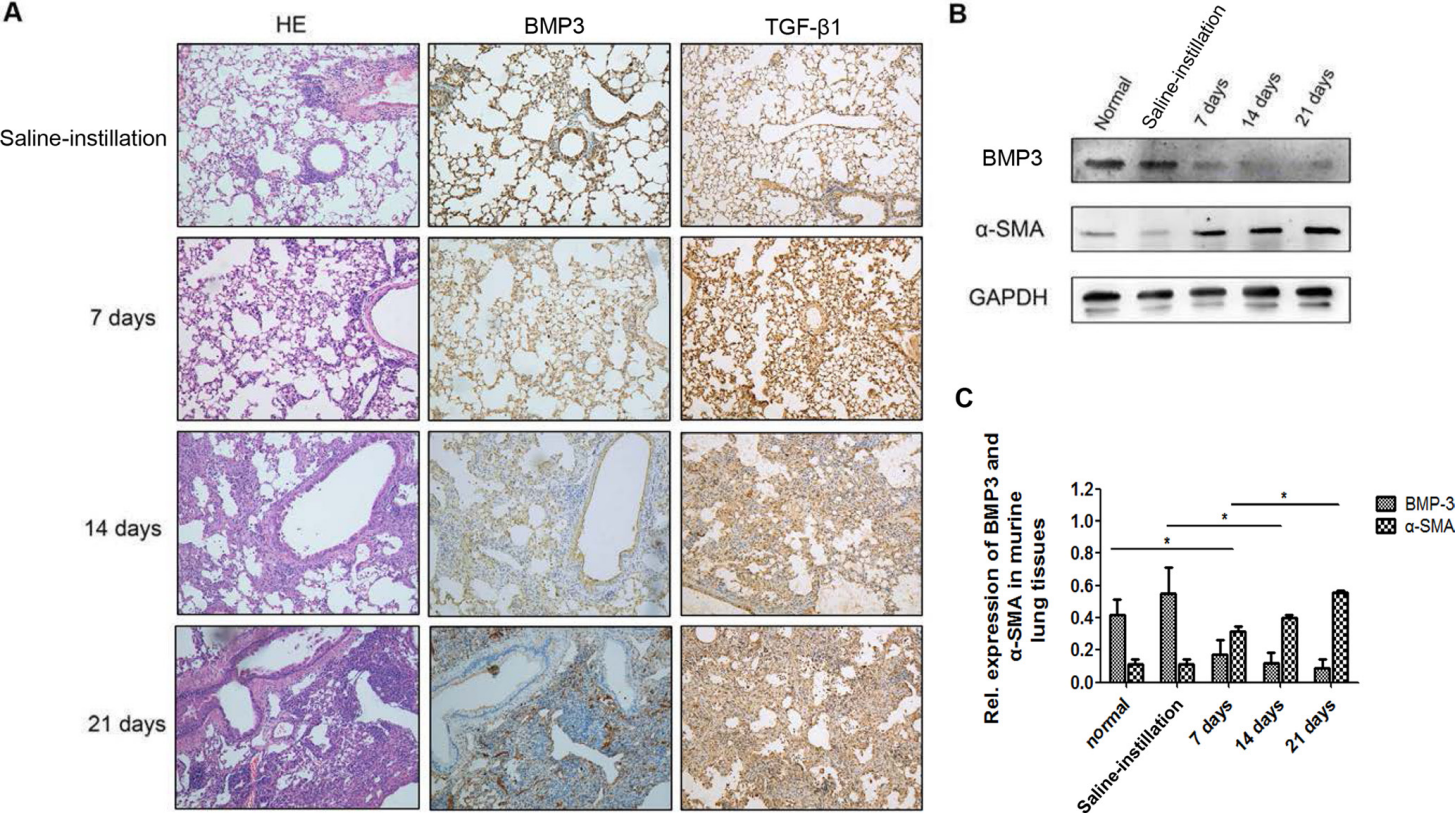
Expression of BMP3 and TGF-β1 protein in fibrotic lungs of the bleomycin-induced murine model of pulmonary fibrosis (**A**) In the saline-instillation group, Bmp3 and TGF-β1 were prominently expressed in bronchial and alveolar epithelial cells. BMP3 expression was noticeably decreased at 7, 14, and 21 days in the bleomycin-induced group as compared with the saline-instillation group, and this altered expression was accompanied by decreased TGF-β1 expression. Notably, strong inflammatory responses were observed at 7 days, and ECM deposition in interstitial space and broadened alveolar septa were observed at 14 days and 21 days in the bleomycin-induced group (*n* = 5 for each group, HE ×200). (**B**) Western blot analysis showed decreased expression of BMP3, while expression of α-SMA, a classic fibrosis marker, was increased concurrently compared to that in normal (untreated) or control-treated (0.9% saline) mice. (**C**) The expression levels of proteins mentioned above were normalized to the expression of GAPDH and quantified using the median immunofluorescence intensity (*P < 0.05). The data were clollected from three independant experiments.

To determine whether BMP3 is a novel factor involved in the pathogenesis of IPF and INSIP as well as a potential new therapeutic target, we established a murine pulmonary fibrosis model based on bleomycin treatment. First, the expression level of BMP3 in mouse lung tissues was measured at different time points after intratracheal instillation of bleomycin. Hematoxylin (HE) staining demonstrated abundant infiltration of lymphocytes into the alveolar space, deposition of extracellular matrix (ECM) in the interstitial space, and substantial widening of the alveolar septa in lung tissues of bleomycin-instilled mice (Figure 2A). In contrast, a minimal inflammatory response was observed in the lung tissues of saline-instillation group (Figure 2A). Importantly, BMP3 expression was markedly reduced in alveolar epithelial and bronchial epithelial cells as well as in interstitial cells in the lungs of bleomycin-treated mice (Figure 2A). To further validate these results, Western blot analysis was performed and demonstrated significantly reduced BMP3 protein levels in 7, 14, and 21 days bleomycin-induced fibrotic lung tissues compared with murine normal lung tissue and saline-treated lung tissues (saline-instillation group) (Figure 2B). In contrast, expression of α-smooth muscle action (α-SMA), a typical marker of myofibroblasts, was significantly increased, demonstrating that bleomycin was rather effective in causing pulmonary fibrosis in mice (Figure 2B). Quantification of Western blots showed that BMP3 expression and the degree of pulmonary fibrosis were inversely correlated (Figure 2C).

### Enhanced BMP3 expression attenuated the fibrotic process triggered by bleomycin in the murine pulmonary fibrosis model

**Figure 3:**
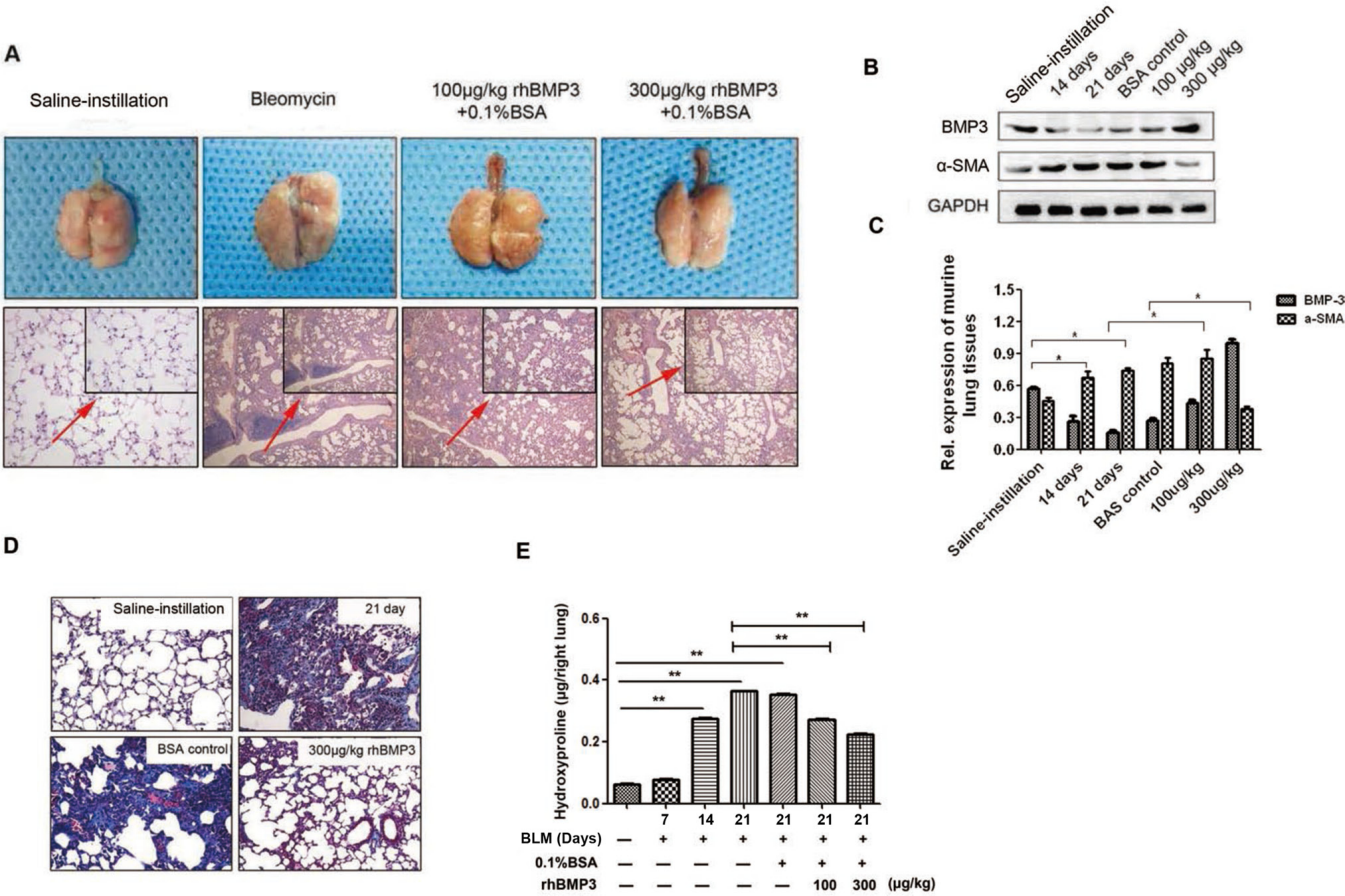
The fibrotic progress was reversed upon BMP3 administration in the bleomycin-induced murine model of pulmonary fibrosis Recombinant human BMP3 (rhBMP3) was injected via the caudal vein at two doses, 100 μg/kg or 300 μg/kg, 7 days after bleomycin treatment.(*n* = 5 for each group) (**A**) Gross anatomical observation showed that lung tissue looked pink and with less inflammation after administration of 300 μg/kg rhBMP3, as compared to the bleomycin treated group (upper panel). Under light microscopy, HE staining revealed that the alveolar interval became significantly narrowed and ECM deposition was reduced following rhBMP3 treatment, especially at the higher dose of BMP3 (lower panel ×100). (**B–C**) Quantification of Western blot analysis of BMP3 and α-SMA expression revealed that α-SMA expression was significantly increased after 14 and 21 days of bleomycin treatment compared with that in saline-instilled mice, and treatment with 300 μg/kg rhBMP3 significantly reduced α-SMA expression compared with that in mice treated with bleomycin (*P <* 0.05), indicating reduced fibrosis following BMP3 treatment. (**D**) Masson's trichrome staining demonstrated that formation of collagen fibrils was augmented after 21 days of bleomycin administration compared with that in saline control group and was reduced back to normal levels following rhBMP3 treatment compared with that in control-treated mice (×100). (**E**) Treatment with rhBMP3 resulted in a decreased hydroxyproline content in the bleomycin-induced murine model of pulmonary fibrosis (*P <* 0.01). Each group has five mice and each experiment was independently three times.

To determine the contribution of reduced BMP3 expression to the pathogenesis of pulmonary fibrosis, a recombinant human (rh) BMP3 was injected via the tail vein of mice 7 days as establishment of the murine pulmonary fibrosis model with bleomycin. Two doses, one low (100 μg/kg) and one high (300 μg/kg) were used. At Day 21, mice were sacrificed, and gross anatomical analyses showed that lung tissues of the bleomycin-only treated group were dark red with evidence of congestion and swelling, whereas lung tissues treated with additional rhBMP3 showed the reduced congestion (Figure 3A). Microscopic images of HE-stained lung tissues revealed substantial lymphocyte infiltration, formation of lymphoid follicles, and a widened interstitial space for the fibrotic lung (Figure 3A). With rhBMP3 treatment the number of inflammatory cells and congestion were reduced (Figure 3A). Thus, the fibrotic pathology was partly reverted in a dose-dependent manner following injection of rhBMP3.

Increased α-SMA expression indicated the conversion of fibroblasts into myofibroblasts, which is accompanied by shrinkage of fibroblasts and secretion of large amounts of collagen, fibronectin, laminin, and other ECM components [37, 38]. Western blot analysis for BMP3 and α-SMA expression confirmed that intravenous injections of rhBMP3 dose-dependently reversed α-SMA expression following bleomycin treatment (Figure 3B–3C). This was indicative of a reversal of the myofibroblasts back to the normal pulmonary fibroblast phenotype. Masson's Trichrome staining demonstrated a substantial increase in collagen deposition in bleomycin-induced mice at Day 21 compared with saline-instillation group. Collagen deposition was significantly decreased with increasing doses of injected rhBMP3. Especially in the high-dose group (300 μg/kg) compared with 0.1% bovine serum albumin (BSA) control-injection group (Figure 3D). In addition, the level of hydroxyproline, a metabolic product of collagen, was measured to indirectly quantify collagen content in lung tissues. Again, rhBMP3 treatment at concentrations of 100 and 300 μg/kg significantly reduced the content of hydroxyproline compared with that in the 0.1% BSA control-injection group at Day 21 following bleomycin-treatment (Figure 3E, *P* < 0.01).

### BMP3 reduced proliferation and activation of primary murine pulmonary fibroblasts

**Figure 4:**
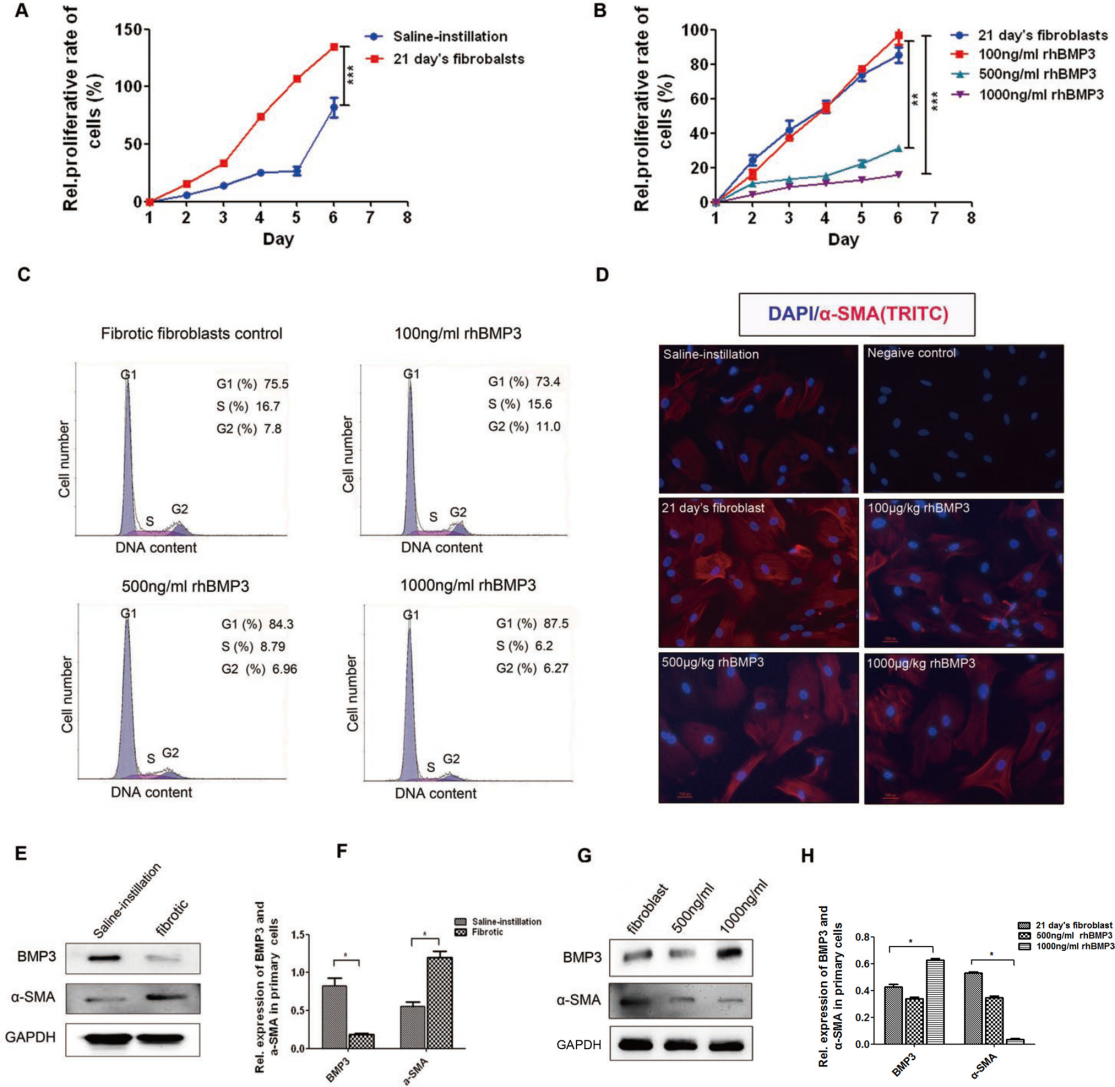
BMP3 reduced proliferation and activation of primary fibroblasts in 21-day bleomycin-induced mice CCK8 was used to detect the proliferation of normal fibroblasts derived from saline-instilled mice and fibrotic fibroblasts derived from BLM-instilled mice with or without stimulation with different concentrations of rhBMP3 for 6 days. (**A**) Fibrotic fibroblasts (21 days) proliferated faster than normal fibroblasts derived from saline-instilled mice, and (**B**) rhBMP3 inhibited the proliferation of fibrotic fibroblasts starting at 500 ng/ml rhBMP3. (**C**) The cell cycle assay showed that the percentage of fibrotic fibroblasts in S phase decreased from 16.7% in control fibroblasts to 15.6%, 8.79%, and 6.2% after treatment with 100 ng/ml, 500 ng/ml, and 1000 ng/ml rhBMP3, respectively. (**D**) Immunofluorescence staining confirmed the changes in α-SMA expression in normal (saline-instilled mice) and fibrotic fibroblasts with or without rhBMP3 stimulation. The number of α-SMA–positive cells was high among fibrotic fibroblasts compared with saline treated fibroblasts. High doses of rhBMP3 (500 and 1000 ng/ml) reduced cell numbers as well as α-SMA immunoreactivity (×200, *P < 0.01). DAPI was used as a nuclear stain. Each experiment was repeated independently three times. (**E–H**) Western blot analysis showed that BMP3 expression was significantly decreased in fibrotic fibroblasts, with a concurrent increase in α-SMA expression (E–F). Administration of rhBMP3 (500 and 1000 ng/ml) decreased expression of the fibrotic marker, α-SMA, (*P < 0.05, **P < 0.01) as normalized to GAPDH expression (G–H).

To investigate the underlying mechanism by which increasing BMP3 could attenuate the progression of pulmonary fibrosis, we isolated primary pulmonary fibroblasts from 21-day bleomycin-induced mice (21 day's fibroblasts) and saline-instilled mice (Normal fibroblasts) according to the method described in a previous study [39]. Isolated cells from BLM lungs were identified as fibroblasts by immunocytochemical staining for α-SMA, vimentin and cytokeratin (Supplementary Figure 2). Cellular proliferation test showed that BLM fibroblasts had a higher proliferative ability than normal fibroblasts (Figure 4A, *P* < 0.05). After administration of rhBMP3, the proliferation rate of BLM fibroblasts decreased in a dose-dependent manner. When the rhBMP3 dose reached 500 ng/ml or 1000 ng/ml, a statistically significant reduction in cell proliferation was observed (Figure 4B, *P* < 0.05). Additionally, the cell cycle progression profiles of BLM fibroblasts with rhBMP3 treatment showed that the majority BLM fibroblasts were arrested in the G1 phase (Figure 4C). The proportion of S phase cells decreased from 16.7% in control fibroblasts to 8.8% and 6.2% after rhBMP3 treatment at doses of 500 ng/ml or 1000 ng/ml, respectively. In consistent with the results *in vivo*, immunofluorescence staining (Figure 4D) and western blot analysis of fibroblast cultures showed higher expression levels of α-SMA and lower levels of BMP3 in BLM fibroblasts compared with normal fibroblasts (Figure 4E–4F), and reduced α-SMA expression following treatment with 500 or 1000 ng/ml rhBMP3 (Figure 4G–4H). These results above indicated that increasing BMP3 levels could inhibit proliferation of primary pulmonary fibroblasts.

### BMP3 prevented the fibrotic process via inhibition of the TGF-β1/Smad signaling pathway

**Figure 5:**
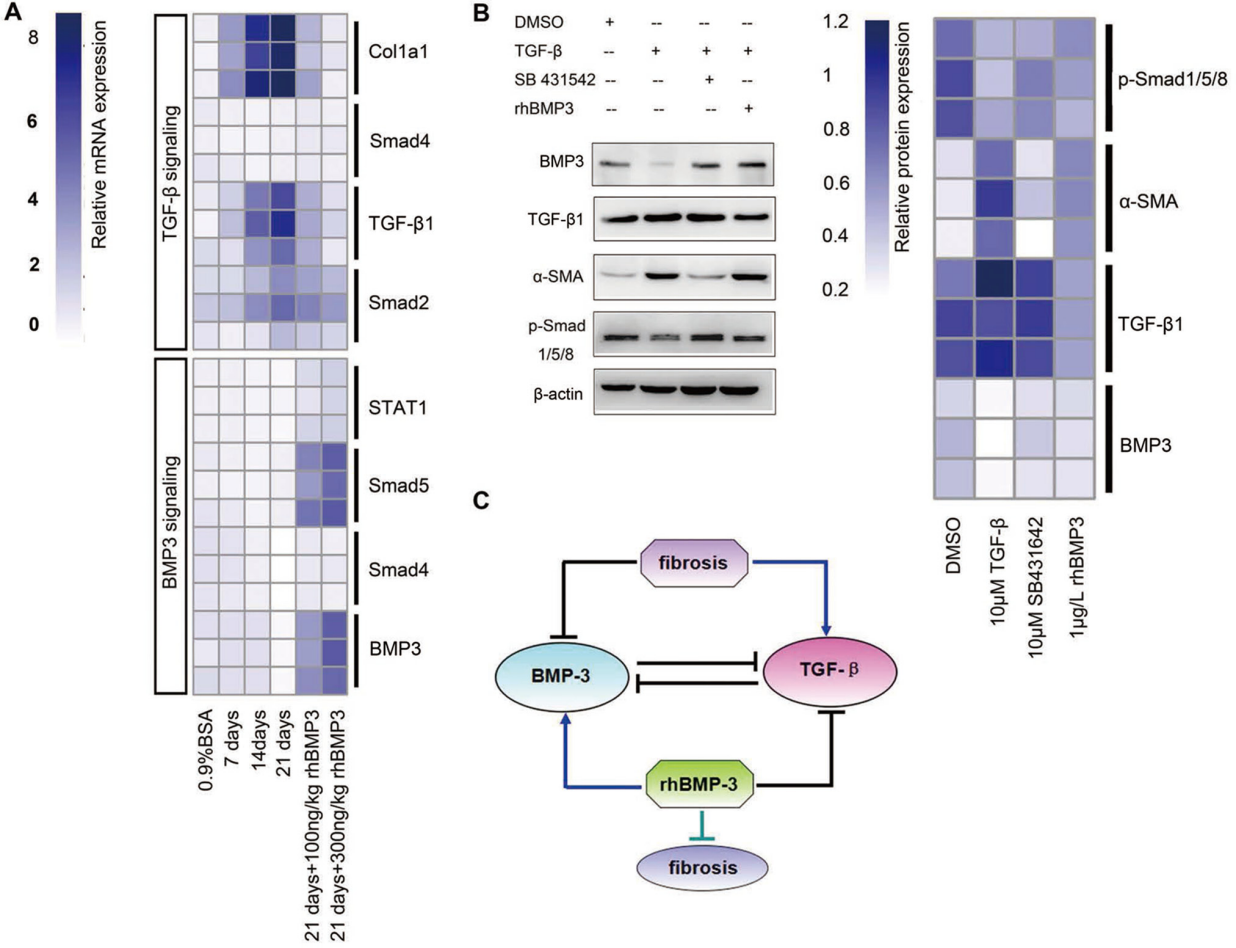
Semi-quantitative RT-PCR revealed the mutually inhibitory relationship between Bmp3 and Tgf-β1 in fibrotic fibroblasts of BLM and NIH-3T3 fibroblasts (**A**) Heat-map showing that the expression of *Tgf-β1* signaling pathway components was up-regulated, whereas the *Bmp3* signaling pathway was down-regulated in BLM. After rhBMP3 stimulation (21 days + 100 ng/ml rhBMP3, 21 days + 500 ng/ml rhBMP3), the expression of *Bmp3* signaling components was increased, which was accompanied by decreased *TGF-β1/Smad* signaling pathway expression compared with control-treatment (0.9% saline). (**B**) Western blot analysis showed the relationship between the TGF-β1 and BMP3 signaling pathways in fibroblasts following treatment with or without the TGF-β1 inhibitor SB431542 or rhBMP3 stimulation. After TGF-β1 administration, BMP3 and p-smad1/5/8 were downregulated, and their expression was rescued by SB431542 and rhBMP3 treatment. The protein expression levels were normalized to the expression of β-actin and quantified according to the mean fluorescence intensity as presented in the heat map. (**C**) Schematic diagram of the relationship between BMP3 and TGF-β1 in the progress of pulmonary fibrosis in BLM. The data were derived from at least three independent experiments.

Previous study demonstrated that TGF-β1 was highly expressed after bleomycin treatment as previously reported [36]. And in this study, TGF-β1 was increased in both RNA sequencing test and immunochemistry staining. Therefore, BMP3 and TGF-β1 expression appeared to be inversely regulated during pulmonary fibrotic process. To delineate the relationship between these two important, seemly opposing factors, quantitative realtime-PCR and Western blot analyses were carried out. The mRNA expression of *Tgf-β1* and its downstream signaling molecules was measured in lung tissues of BLM-instilled mice with or without rhBMP3 treatment. *Tgf-β1*, *Smad2*, and *Col1α1* mRNA expressions were increased in BLM-induced pulmonary fibrosis, whereas they were decreased after rhBMP3 administration in a dose-dependent manner (Figure 5A and 5B). Interestingly, *Smad4* expression showed no obvious difference across all conditions (Figure 5A). As expected, the expressions of *Bmp3* as well as its downstream signaling molecules *Smad5* and *Stat1* were significantly increased with rhBMP3 treatment, indicating that injected rhBMP3 was functional and the BMP3 and TGF-β1 pathways were mutually antagonizing (Figure 5C).

Given that administration of rhBMP3 reduced TGF-β1 expression, the question arose whether the converse treatment with TGF-β1 would down-regulate BMP3 expression. Therefore, the murine fibroblast cell line NIH3T3 was treated with TGF-β1 with or without its antagonist SB431542, or with rhBMP3 (Figure 5B). After TGF-β1 stimulation, NIH3T3 cells became mitotically active (Supplementary Figure 3), and the expression of BMP3 was significantly reduced, which was accompanied by a decrease in expression of BMP3's downstream signaling factors phospho-Smad1/5/8 (p-Smad1/5/8) and a concurrent increase in α-SMA (Figure 5B). The TGF-β1 receptor (Alk5)-specific inhibitor SB431542 strongly counteracted the effect of TGF-β1 on BMP3 expression, without affecting TGF-β1 expression. In consistent with the outcome by SB431542 administration, rhBMP3 application also reversed the TGF-β1 effect (Figure 5B).

Taken together, these results suggested that BMP3 may alleviate the fibrotic processing by suppressing the activation of TGF-β1 signaling pathway and that the mutually antagonistic relationship between BMP3 and TGF-β1 plays a crucial role in the pathogenesis of pulmonary fibrosis (Figure 5C).

Reduced BMP3 expression was associated with worse survival rate in IPF patients To further determine the clinical importance of our finding regarding the role of BMP3 in the pathogenesis of pulmonary fibrosis, BMP3 expression was examined using tissue array technology and immunohistochemistry in 47 cases of IPF and INSIP patients with more than 5 years of detailed clinical follow-up data including in 83 IIP cases (Supplementary Table 2, Supplementary Figure 4A).

**Figure 6:**
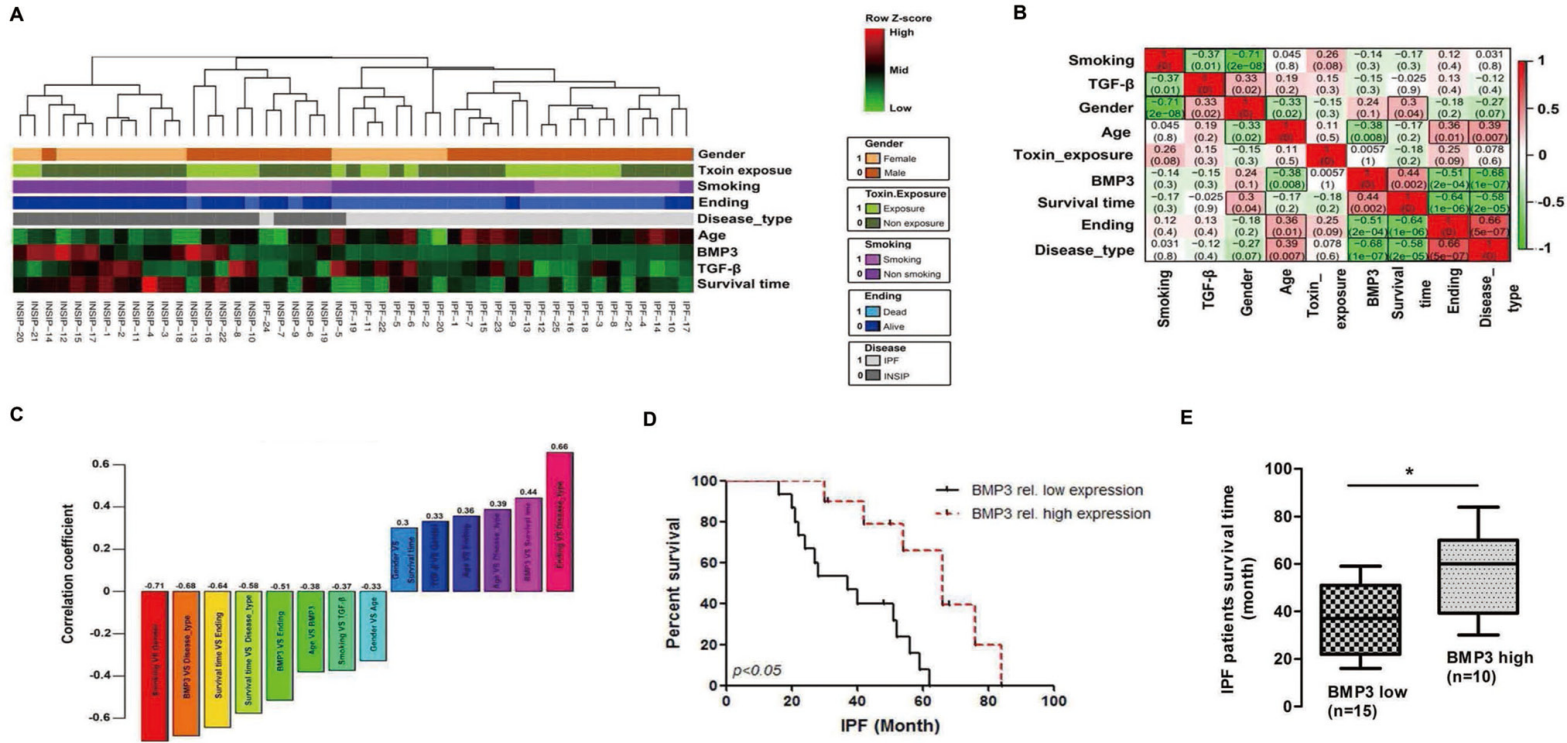
High BMP3 expression predicted better survival rate in IIP patients (**A–B**) Hierarchical clustering and correlation coefficients of eight parameters in all 47 IIP patients. (**C**) Pairwise correlation coefficient of eight parameters in all combinations with *P* values < 0.05. (**D–E**) Survival curves and survival times of IPF patients with relatively high or low BMP3 expression (*P < 0.05).

A more than 5-year clinical follow-up of 22 INSIP and 25 IPF patients provided a data matrix of eight parameters including gender, age at disease diagnosis, smoking, chronic toxin exposure, survival time, whether the patient was deceased (death), as well as the levels of TGF-β1 and BMP3 proteins in lung tissue biopsy specimens. The data matrix with these eight parameters plus the disease type (INSIP or IPF) was subjected to hierarchical clustering, and the Pearson's correlation coefficients between any random pairs of these parameters were calculated (Figure 6A–6C). It is known that INSIP patients survive longer than those with IPF, and accordingly, disease type was correlated positively with death and negatively with survival time (IPF was designated as 1 and INSIP as 0 to digitize the disease type parameter). Most of the smokers in this patient cohort were male, and thus, gender was negatively correlated with smoking (correlation coefficient: -0.71). Interestingly, smoking was weakly but significantly correlated with TGF-β1 expression (correlation coefficient: -0.37). Therefore, smokers exhibited lower TGF-β1 levels in their diseased lung tissues. TGF-β1 was also weakly correlated with gender, probably because gender and smoking were tightly correlated in this particular data set. Age was weakly correlated with gender, death, disease type, and BMP3, probably due to the fact that the IPF patient pool was older than the INSIP patient pool, which was consistent with the clinical observation that INSIP occurred more frequently in younger people as compared to IPF (5). BMP3 was found to be expressed at low levels in patients with IPF, which presented as a more severe disease with higher lethality as compared to INSIP. Consequently, BMP3 expression was correlated negatively with death and age, and positively with survival time (Figure 6B and 6C).

It became obvious that IPF patients who expressed below the average levels of BMP3 survived shorter times than those who expressed above the average levels (Figure 6D and E, p = 0.006). Most INSIP patients had higher BMP3 expression levels and consequently showed overall longer survival times. This result was consistent with results showing that high BMP3 expression prolonged survival time in INSIP patients (Supplementary Figure 4B). In contrast, TGF-β1 expression did not predict prognosis in either IPF and INSIP patients regardless of low or high, TGF-β1 expression levels (Supplementary Figure 4C and 4D). Taken together, these results indicate that BMP3 may be a beneficial factor for predicting prognosis in both IPF and INSIP patients. Particularly in IPF, elevated BMP3 expression can serve as a potential treatment strategies for IIP.

## DISCUSSION

IIPs, either IPF or INSIP, are always associated with fibrosis, but the underlying pathological mechanisms are under investigation. In order to identify genes that are dysregulated in lung tissues from IPF and INSIP patients, RNA-sequencing of patient lung tissues combined with Weighted Gene Co-expression Network Analyses (WGCNA) was employed as a very powerful bioinformatics tool for transcriptome analyses. This approach identified dramatic changes in the expression of not only individual genes but also of functionally relevant gene clusters/modules that were positively and negatively correlated with IPF/INSIP. *TGF-β1*, representing positive cell cycle regulation, was found to be upregulated, whereas *BMP2*, representing negative regulation of cell cycle, and *BMP3*, representing crucial cell–cell signaling, were downregulated in IPF/INSIP. Since BMP3 has not been previously reported to be involved in IIPs, an extensive series of *in vivo* and *in vitro* studies were carried out using the bleomycin-induced pulmonary fibrosis animal model. These experiments demonstrated that a decrease in BMP3 expression not only served as a biomarker for IPF, but that BMP3 *per se* was a functionally relevant factor. Manipulation of BMP3 expression significantly attenuated the fibrotic process following BLM. Moreover, the *in vitro* cell culture studies with normal and fibrotic murine fibroblasts further demonstrated that, like BMP2 [40, 41], BMP3 also functionally antagonized TGF-β1 to reduce fibrotic cell proliferation.

Balanced TGF-β and BMP signaling has been proposed to be crucial for the progression of fibrosis [42]. Meng et al. suggested that TGF-β1 is activated upon fibrosis, disrupting the balance between TGF-β1 and BMP7 resulted in the suppression of BMP7 expression and its target genes [33, 43]. Similarly, in the present study, a mutually inhibitory relationship between TGF-β1 and BMP3 was observed in both human patients and a murine fibrosis model. Our results showed that regardless of whether TGF-β1 was up- or down-regulated, it affected the expression of BMP3 signaling in a converse manner. Moreover, BMP3 also affected the TGF-β1 signaling pathway and TGF-β1 expression.

One of the strengths of this study was the use of clinical data including more than 5 years of clinical follow-up of 25 IPF and 22 INSIP patients. Although some BMPs have been discovered as anti-fibrosis factors, the involvement of BMPs in the pathogenesis of pulmonary fibrosis in humans, and the relationship between BMPs and disease progression of IIPs remains largely unknown [44–47]. By using patient clinical information together with quantified BMP3 and TGF-β1 levels from lung biopsies, the present study confirmed that BMP3 was negatively correlated with disease severity. Surprisingly, TGF-β1 was not statistically significant associated with patients’ survival time but showed a trend of positive correlation with disease severity. It is likely that analysis with increased patient numbers will show the TGF-β1 is a reliable predictor of disease severity. Nevertheless, when comparing TGF-β1 and BMP3, the latter was significantly associated with survival time of IPF patients in the present study. This is likely because BMP3 is more directly linked to IIPs, whereas TGF-β1 might be linked somewhat more indirectly, as its expression can be regulated by a variety of factors, including lung and immune cells.

BMP3 was expressed at higher levels in INSIP lungs compared with IPF lungs, consistent with its beneficial role, as INSIP is known to be a less severe clinical condition than IPF. However, in INSIP, BMP3 downregulation was a minor event and did not significantly impact the disease outcome. In IPF on the other hand, because patients displayed much more reduced BMP3 levels, variations in BMP3 levels served as a good indicator for survival rates. In the future, larger patient cohorts need to be analyzed for BMP3 expression levels to confirm the role of BMP3 as a clinically relevant predictor of disease prognosis, particularly for IPF. Given that in animal studies BMP3 showed protective effects, it also will be interesting to determine whether enhancing BMP3 protein levels might serve as a therapeutic strategy for IPF.

In summary, we identified that BMP3 plays a protective role in fibrosis and is a valuable anti-fibrotic factor for IPF. Although the number of samples used in the initial RNA sequencing analysis was relatively low, we further confirmed the expression of BMP3 in the lung tissues of 83 patients with IIPs confirmed by clinical-radiologic-pathological diagnosis. Importantly, more than 5 years of follow-up clinical data of 47 cases including in 83 cases were available, which strongly supported that BMP3 might be a novel biomarker in IPF and INSIP, and especially IPF. We also showed that BMP3 could suppress the fibrotic process of pulmonary fibrosis both *in vivo* and *in vitro*. Finally, our results uncovered an antagonistic relationship between BMP3 and TGF-β1, which played a critical role in the pathogenesis of pulmonary fibrosis in BLM. Our study highlights the potential clinical value of BMP3 for the treatment of IPF and INSIP patients in the China population.

## MATERIALS AND METHODS

### Study design

Clinical data of 83 patients including 46 IPF cases and 37 INSIP cases were collected from Tongji Hospital affiliated with the Tongji University School of Medicine (Shanghai) and the Guangzhou Institution of Respiratory Diseases, (Guangzhou, China). The detail clinical data of these patients are summarized in Supplementary Table 1. All biopsy samples from IIP patients were collected at initial diagnosis and taken by video-assisted thoracoscopic surgery (VATS) or a small incision. Control samples were collected from normal lung tissue adjacent to the benign pulmonary tumors without fibrosis. Among the 83 IIPs cases, the lung biopsy samples of seven most typical cases of IIP patients were used for RNA sequencing, including four cases of IPF and three cases of INSIP. Correspondingly, 5 normal lung tissues were randomly chosen for RNA sequencing. The expression of BMP3 and TGF-β was confirmed by immunohistochemistry in all tissue samples, including 47 specimens (25 cases of IPF and 22 cases of INSIP) with more than five years follow-up data, which were used to analyze the clinical relevance of these genes, the detail information of 47 cases are summarized in Supplementary Table 2. The criteria for all patients inclusion were: (i) a diagnosis according to the criteria of the American Thoracic Society (ATS)/European Respiratory Society (ERS) classification guidelines on IIPs [3, 30–32]; (ii) availability of integrated clinical, radiologic, and pathologic information; and (iii) a final diagnosis made by pathologists, clinicians and radiologists through multi-disciplinary discussion with all other known causes of ILD excluded.

For *in vivo* experiments in the bleomycin-induced murine pulmonary fibrosis model, at least five mice were randomly selected per group. All analyses were performed blinded to treatment, and all experiments were repeated independently three times.

### Study approval

The use of patient biopsy specimens was approved by the Ethics Committee of the Tongji Hospital [(Tong) Ethics Committee Approval No. 183]. All patients provided informed consent prior to inclusion in the study. All methods were performed in accordance with the Tongji Hospital Center animal care and use committee.

### Bleomycin-induced murine pulmonary fibrosis model

Ninety male C57BL/6J mice (8 weeks old) were purchased from Shanghai Slac Laboratory Animal Co., Ltd (Shanghai, China). All animals were housed under specific pathogen-free conditions at the Laboratory Animal Care-approved facility of the Tongji Hospital. Mice were randomly assigned to different groups, which were either treated with 5 mg/kg of bleomycin (*n* = 50, 1 mg dissolved in 1 ml of saline; Tokyo, Japan) or saline only (*n* = 30) (Shanghai Slac Laboratory Animal Co.). Bleomycin-treated mice were injected with 100 mg/kg rhBMP3 (*n* = 15, 0.1% BSA in saline according to the manufacturer's instructions, PeproTech, Rocky Hill, NJ, USA), 300 mg/kg rhBMP3 (*n* = 15), or 0.1% BSA only (*n* = 15) through the caudal vein every other day after the initial 7 days of bleomycin induction and until lung harvest. Additionally, a group of naïve mice were sacrificed without any injection (*n* = 10). The tissues were harvested at Day 7, 14 and 21 after bleomycin or saline treatment. Mice were anaesthetized by overdose pentobarbital and were infused with 30 ml saline via left ventricular before death. The right lungs were frozen immediately in liquid nitrogen for total RNA or protein extraction or the hydroxyproline assay, whereas the left lungs were soaked in 4% PFA for overnight and were embedded in paraffin for morphological observations (HE and Masson's trichrome stain).

### Library construction and RNA sequencing

Total RNA from 10 mg tissue was isolated with depleting genomic DNA for RNA-seq library construction following standard TruSeq RNA sample preparation v2 protocol (Illumina, San Diego, CA, USA). The sequencing libraries were then sequenced using the Illumina HiSeq2000 platform. From reads averaging 50 bp in length, 9.0 ± 1.5 million reads per sample were generated. After that, the reads were aligned to the human reference genome (GrCH37, Ensembl build 74) using Tophat version 2.0.12, yielding an average mapping rate of 94.8 ± 2.1%. Gene expression levels, which were represented as fragments per kilo-base per million mapped reads (FPKM), were obtained for 63,783 genes/transcripts annotated using Ensemble GrCH37 database release 74. The RNA-sequencing data were deposited to the Gene Expression Omnibus (GEO) repository (accession number GSE73189).

### Semi-quantitative reverse transcription PCR (RT-PCR) and real-time PCR

Total RNA was extracted by using TRIzol reagent (Invitrogen Life Sciences; Carlsbad, CA, USA) following the manufacturer's instructions. Total RNA was transcribed into cDNA by PrimeScript^TM^ RT Master Mix kit (Takara Bio Company). Real-time PCR was performed using SYBR Premix EX TaqTM kit (Takara). The details of primer sequences for mouse *Bmp3, Tgf-β1, Smad4, Smad2, Smad3, Smad1, Col1a1, α-SMA, and Stat1* are listed in Supplementary Table 3.

### Bioinformatics analysis

Gene ontology enrichment analysis was carried out by a Bioconductor package topGO. KEGG pathway enrichment analysis was carried out by a Bioconductor package GSEABase (http://www.r-project.org/). Terms were accepted if they hit more than one gene and a Fisher's Exact Test *P* value of < 0.05.

### Weighted gene co-expression analysis (WGCNA)

For WGCNA, 5156 transcripts that were significantly differentially expressed between the two groups were selected (Tukey's HSD test with *P* value < 0.05). A weighted gene co-expression network was constructed, and a matrix of signed Pearson correlation coefficients between all pairs of transcripts was computed. This correlation matrix was raised to power β = 12, which was the default parameter for WGCNA to calculate an adjacency matrix. To minimize the noise and spurious associations, the adjacency matrix was transformed to topological overlap matrix (TOM). The matrix 1-TOM was used as the input of average linkage hierarchical clustering, and genes with similar expression patterns were clustered together. The expression profile of a given module was represented by its first principal component (known as module eigengene, ME), which could represent the expression levels of the overall module. For module-trait correlation calculation, all factorial traits were convert to numeric vector based on its factor levels. Module-sample type correlation was calculated by correlating the ME of each module to the sample type model. Immunohistochemical staining The lung biopsies of 83 patients were performed immunohistochemical staining to determine the expression of BMP3 and TGF-β. Among these patients, the biopsies of 47 cases were used to establish the tissue arrays following a method previously described (Shanghai Outdo Biotech Co. Ltd) [33], which were possess the integrated five-years following-up data. Briefly, tissue arrays were constructed from 154 tissue samples collected from 47 lung thoracoscopic surgery specimens of patients with IPF and INSIP (Each specimen was randomly taken 3 samples to construct tissue arrays), and 13 normal tissue specimens (Each specimen was randomly taken 1 sample for construction). After epitope de-masking, tissue arrays and the other lung biopsies of 36 patients were immunostained with antibodies against BMP3 (affinity-purified rabbit polyclonal IgG; Santa Cruz Biotechnology Inc.; Santa Cruz, CA, USA) and TGF-β1 (affinity-purified rabbit polyclonal IgG; Cell Signaling Technology; Danvers, MA, USA) overnight at 4°C. Staining was then detected with affinity-purified, biotinylated secondary antibodies (Dako Cytomation; Ely, UK). Staining was visualized with the DAKO Envision System. Each stained section was randomly captured in at least five different fields at 400× magnification. The images were used to estimate the mean optical density (OD) by two specialists in the field of lung pathology with Image pro plus software (Media Cybernetics, Rockville, MD, USA) under blinded conditions using the following formula: mean OD = sum of total integrity OD/total area.

### Hydroxyproline assay

The hydroxyproline content per 100 mg of lung tissue was measured as an index of lung fibrosis using a Hydroxyproline Assay Kit (KeyGen Biotech; Nanjing, China). Briefly, Lung tissues were cut into small fragments and hydrolysis in 6N HCL at 95°C by 5 hours. The hydroxyproline was then separated from powdered activated carbon by centrifugation and supernatant of each sample was incubated as the instruction with solutions supplied by the kit. After the incubation, the suspension was centrifuged at 3500rpm for 5 minutes. The supernatant OD was read under 550nm wavelength of light and hydroxyproline concentrations calculated from standard curve. The formula was hydroxyproline content (*ug/mg*) = (sample's OD-blank control's OD) / (standard's OD-blank control's OD) x 5ug/ml x total volume of hydrolysis (*10ml*) /weight of tissue (*mg*).

### Isolation and treatment of murine primary lung fibroblasts

Adult rodent lung fibroblasts were isolated from C57B/6J mice that were or were not treated with bleomycin as described previously [34]. Cells were cultured in DMEM/F12 with 15% FBS and antibiotics/antimycotics at 37°C in 5% CO_2_.

### Cell proliferation and cell cycle assays

Cultured fibroblasts (1×10^3^/well) were seeded in 96-well plates for 24 hours and exposed to different concentrations of rhBMP3 for 1–6 days. Cellular proliferation was detected using a tetrazolium salt-based colorimetric assay kit (CCK8 kit, KeyGen Biotech, Nanjing, China), as previously described [35].

The cell cycle was estimated by flow cytometry using a cell cycle and apoptosis analysis kit (Beyotime, China). Briefly, Amount of 1×10^6^ cells were digested by trypsin and the suspension was centrifuged at 1000×*g* for 5 minutes. After washing the sediment with cool PBS, the cells were fixed in cool 70% ethanol at 4°C for 12 hours. Propidium Iodide (PI) and RNase A were added in cell suspension according to instructions and incubated at 37°C for 30 minutes. After staining, samples were tested under 488nm excitation wavelength of light. Data were analyzed by homologous software Flowjo 7.6 (FlowJo LLC, Oregon, USA).

### Western blot analysis

Total protein was extracted from lung homogenates or cell lysates using radioimmunoprecipitation (RIPA) buffer containing phenylmethylsulfonyl fluoride (PMSF) and phosphatase inhibitors as previously described [35]. After electrophoresis and membrane transfer, the immunoblots were probed with the following primary antibodies: BMP3 (1:200, Santa Cruz Biotechnology), phosphor-Smad1/5/8, Smad4 (1:1000, Cell Signaling Technology; Beverly, MA, USA), α-SMA (1:200, Dako), GAPDH, and β-actin (1:5000, Bioworld, Dublin, OH, USA). The secondary antibodies were goat anti-rabbit or goat anti-mouse horseradish peroxidase-conjugated antibody (Cell Signaling Technology). Enhanced chemiluminescence reagent was used for detection and blots were scanned using the Alphaview SA software (Proteinsimple, San Jose, CA, USA).

### Immunofluorescence staining

Cells were cultured on lysine-treated slides in a 6-well chamber prior to immunofluorescent staining. The cells were fixed in 4% paraformaldehyde for 15 min at room temperature. After washing with PBS, cells were permeabilized with 0.1% Triton X-100 for 30 min and blocked with 10% BSA in phosphate-buffered saline (PBS) for 60 min in a humidified chamber. Cells were incubated with α-SMA antibody (1:200 dilutions, Dako) overnight at 4°C. After washing with PBS, cells were incubated with TRITC-conjugated secondary antibody. Cell nuclei were counterstained with DAPI (Beyotime Biotechnology). Phase contrast and fluorescent microscopy was performed using an Olympus IX81 inverted research microscope (Olympus; Tokyo, Japan).

### Statistical analysis

Statistical analysis was performed using the SPSS software program, version 19.0 (IBM Corp. Released 2010. Statistical differences among groups were determined by one-way analysis of variance (ANOVA) followed by Tukey's test for qPCR, mean integrity OD, and hydroxyproline measurements. Survival functions were evaluated by Kaplan–Meier survival analysis. The results are expressed as means ± standard deviation (SD). A probability level of *P* < 0.05 was regarded as statistical significant.

## SUPPLEMENTARY MATERIALS FIGURES AND TABLES



## Abbreviations

IPF: Idiopathic pulmonary fibrosis
INSIP: idiopathic nonspecific interstitial pneumonia
IIPs: Idiopathic interstitial pneumonias
ILs: interleukins
TGF-β: transforming growth factor-β
BMP3: Bone morphogenetic proteins 3
EMT: epithelial-mesenchymal transition
FPKM: fragments per kilo-base per million
WGCNA: weighted gene co-expression network analyses
HE: Hematoxylin
ECM: extracellular matrix
α-SMA: α-smooth muscle action
BSA: bovine serum albumin
BLM: bleomycin-induced mice
