# Supplementary material for: Reduced expression of BMP3 contributes to the development of pulmonary fibrosis and predicts the unfavorable prognosis in IIP patients
Source: Oncotarget. 2017 Aug 9;8(46):80531–44. doi: 10.18632/oncotarget.20083 (PMC5655218; doi:10.18632/oncotarget.20083)
Supplement: Supplementary file 1 [file oncotarget-08-80531-s001.pdf]

Reduced expression of BMP3 contributes to the development of pulmonary fibrosis and predicts the unfavorable prognosis in IIP patients

SUPPLEMENTARY MATERIALS

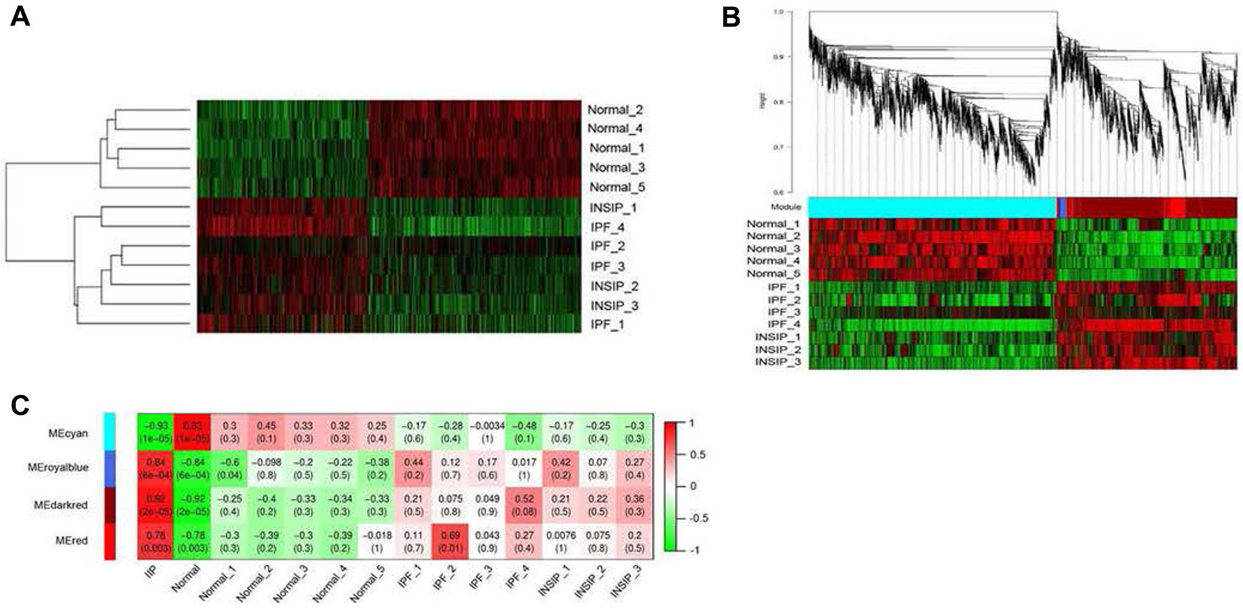

Supplementary Figure 1: Bioinformatics analysis of RNA-seq data. (**A**) Genes that differentiate IPF and INSIP patients with pulmonary fibrosis from normal control subjects are shown. Average linkage clustering with Euclidean distance metric was used. (**B**) Weighted gene co-expression network analysis (WGCNA) of differentially expressed transcripts between normal and IPF and INSIP patient cohorts revealed four co-expression modules. Dendrogram on top shows the hierarchical clustering of the transcripts. The color bands immediately beneath the dendrogram indicate the assigned gene modules. The heat map under the color bands represents the scaled gene expression levels within each module across the 12 samples (high: red, low: green). (**C**) Module-sample, or sample type correlation plots. Correlation value and *P*-values (in parentheses) are presented within each square.

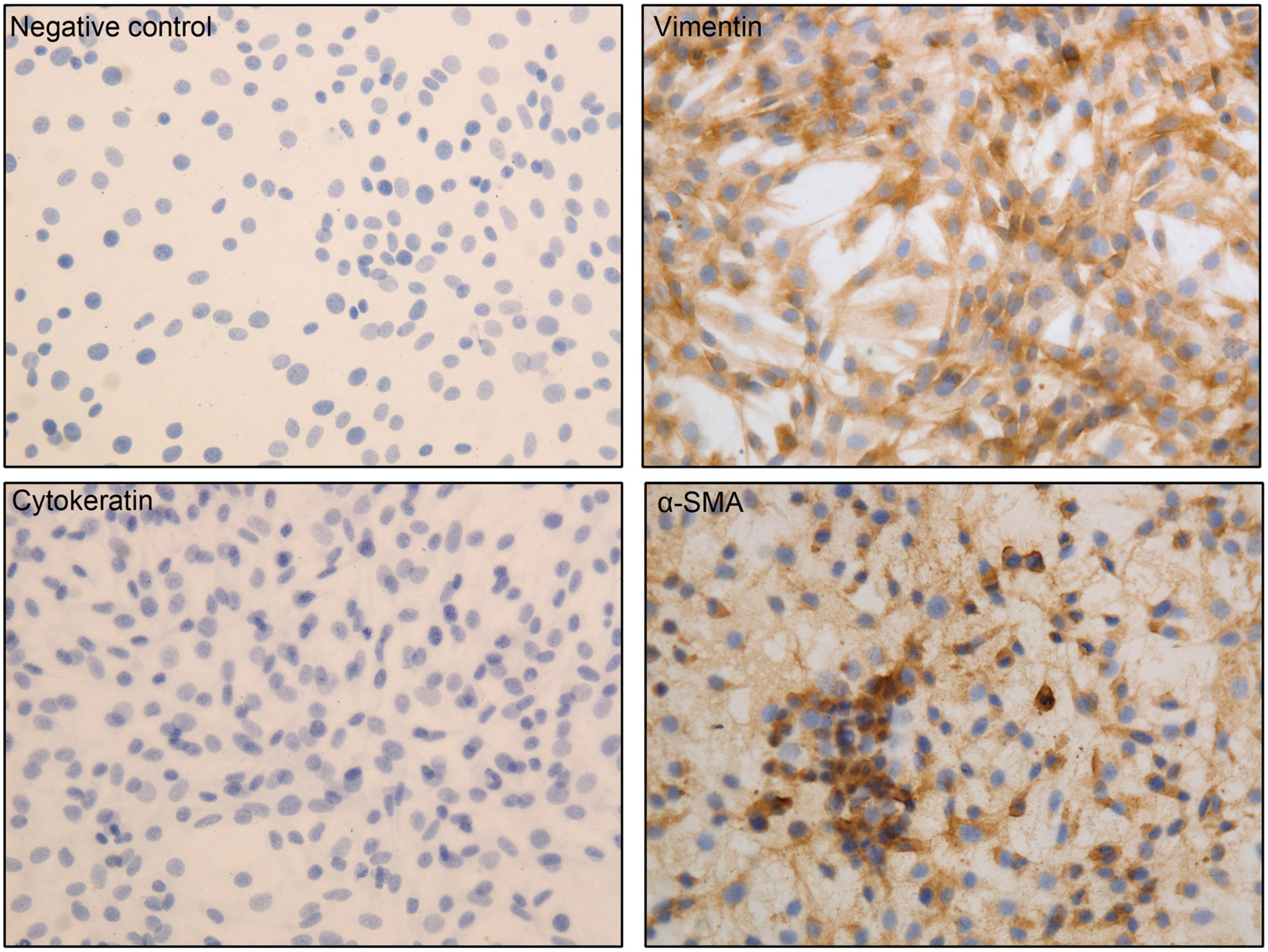

Supplementary Figure 2: Immunocytochemical characterization of primary fibrotic fibroblasts in the bleomycin-induced murine pulmonary fibrosis model. Primary fibrotic fibroblasts isolated from bleomycin-induced murine lung tissues were strongly positive for α-SMA and vimentin, typical myofibroblast and mesenchymal cell markers. In contrast, cytokeratin was not expressed as compared to the level in the negative control group, which was not stained with primary antibodies (Envision, ×100).

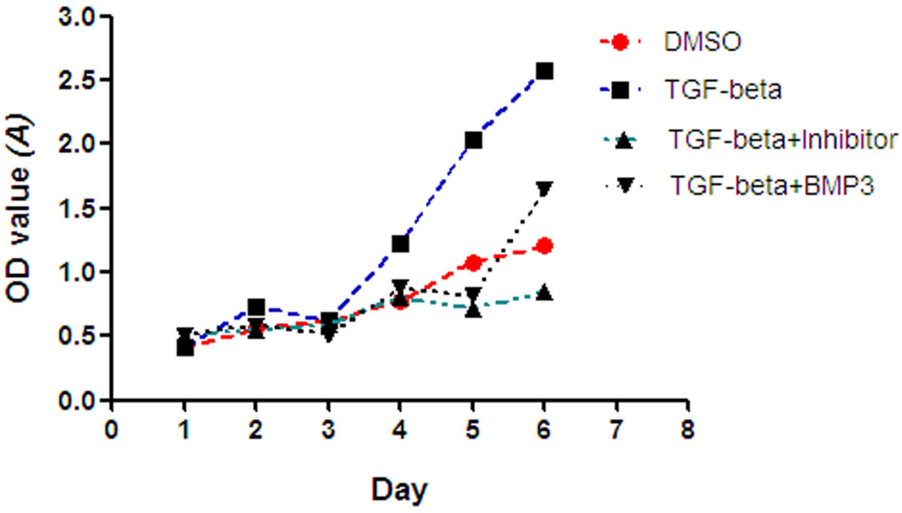

Supplementary Figure 3: Proliferation of murine NIH3T3 fibroblasts treated with TGF-β, with or without its antagonist (SB431542), or BMP3. With TGF-β1 stimulation, the proliferation of NIH3T3 cells was significantly higher than that in the DMSO control group. TGF-β1–induced cells were treated with the TGF-β1 antagonist SB431542 and rhBMP3 12 h after plating.

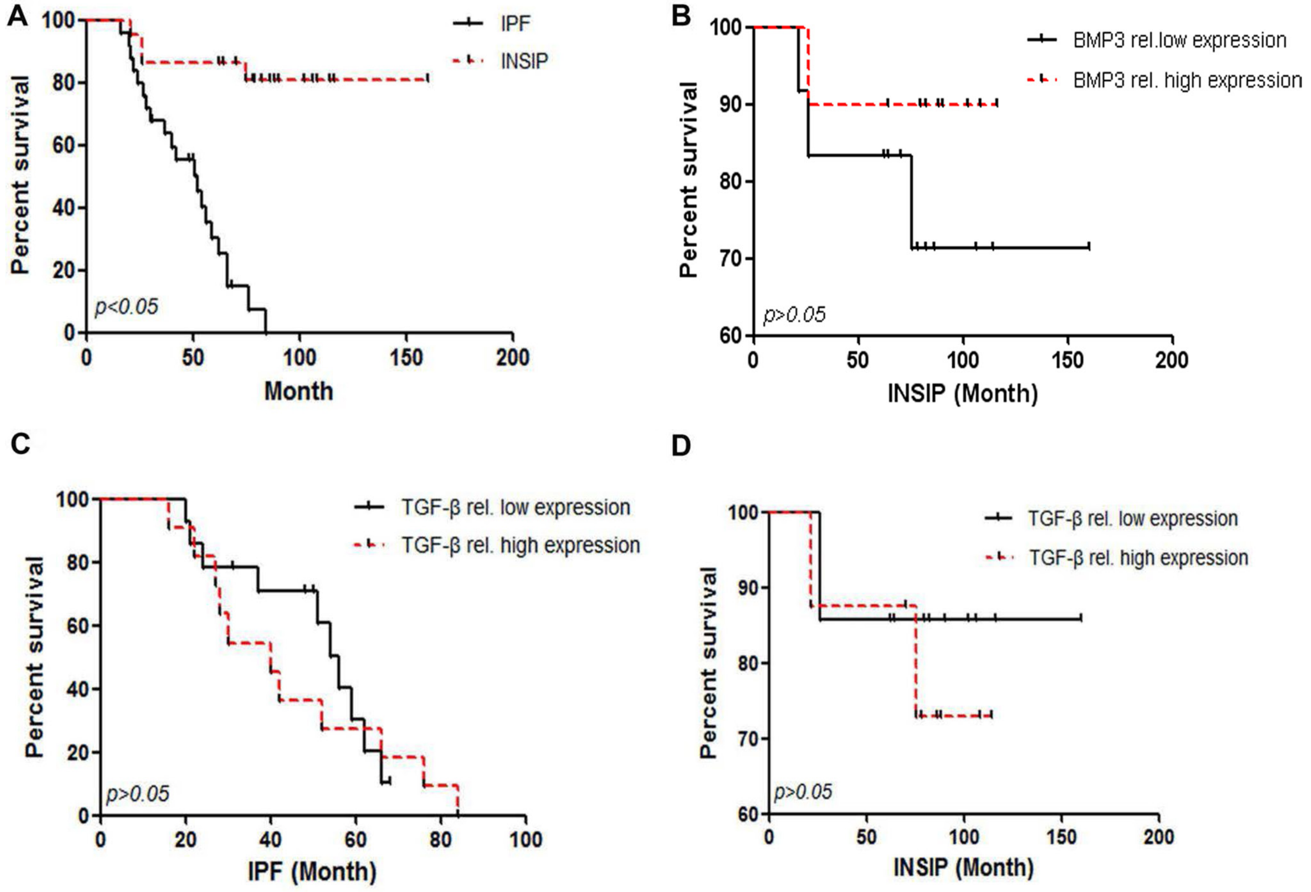

Supplementary Figure 4: Survival curves of IPF and INSIP patients. (**A**) Survival curves (% survival) of patients with IPF or INSIP. (**B**) Survival curves (% survival) of INSIP patients with relatively low or high expression of BMP3. (**C–D**) Survival curves (% survival) of IPF and INSIP patients with relatively low or high expression of TGF-β1.

Supplementary Table 1: Clinical features of 83 IIP patients

Feature	IPF (*n* = 46)	INSIP (*n* = 37)
**Age (yrs)**	56.8 ± 12.3	50.4 ± 8.8
**Median age**	61.0	50.0
**Range**	15-73	37–74
**Gender**		
**Male (%)**	35 (76.1%)	19 (51.4%)
**Female (%)**	11 (23.9%)	18 (48.7%)
**Smoking history (%)**	17 (37.0%)	15 (40.5%)
**Dust exposure history (%)**	12 (16.1%)	8 (21.6%)
**Symptom**		
**Anhelation after activity (%)**	38 (82.6%)	23 (62.2%)
**Velcro (%)**	40 (87.0%)	18 (48.6%)
**Clubbing (%)**	19 (41.3%)	5 (13.5%)

Eight parameters of patients with IPF and INSIP.

Supplementary Table 2: Clinical features of 47 patients with IPF or INSIP

Feature	IPF (*n* = 25)	INSIP (*n* = 22)	t / χ2/ *Kruskal-Wallis*	*P*
**Age (yrs)**	58.2±10.7	50.3±8	2.823	0.007
**Median age**	58.5	50.5		
**Range**	29-75	37-71		
**Gender**			2.433	0.119
**Male (%)**	17 (68%)	10 (45%)		
**Female (%)**	8 (32%)	12 (54%)		
**Smoking history (%)**	10 (40%)	9 (40.9%)	0.004	0.949
**Dust exposure history (%)**	9 (36%)	8 (36.4%)	0.001	0.979
**Symptom**				
**Anhelation after activity (%)**	23 (92%)	16 (72.7%)	1.864	0.172
**Velcro (%)**	24 (96%)	15 (68.2%)	4.593	0.032
**Clubbing (%)**	13 (52%)	4 (18.2%)	5.797	0.016
**Course of disease (month)**	43.64 ± 19.6	80.95 ± 31.8	4.877	0.000
**Follow-up time (month)**	26.6 ± 17.4	69.8 ± 35.1		
**Follow-up results (%)**				
**Improvement/Stability**	0 (0%)	10 (45.4%)		
**Relapse/Exacerbation**	5 (20%)	8 (36.4%)	20.664	0.000
**Death**	20 (80%)	4 (18.2%)		

Ten parameters of patients with IPF and INSIP (**P* < 0.01, ***P* < 0.001).
Definition of abbreviations:IPF= idiopathic pulmonary fibrosis; INSIP = idiopathic nonspecific interstitial pneumonia.

Supplementary Table 3: PCR primer sequences

Gene	Forward (5′→3′)	Reverse (5′→3′)	Length (bp)
BMP-3	AACGATGCTGCCATTTCTG	GCGTCTGTCCCTTCTGATG	313
TGF-β1	TGGAGCCTGGACACACAGTA	GTAGTAGACGATGGGCAGTGG	120
Smad2	ATCACGCCTTGGTTGTCAGT	TCTTGGCAGAGGTAGGGAAG	120
Smad5	CTATGAGGAGCCCAAACACTG	CAACAATCCCAGGCAGAATC	151
Smad4	AGCCTCCCATTTCCAATCAT	CCATCCACAGTCACAACAGG	124
Col I a1	CTGACTGGAAGAGCGGAGAG	GACGGCTGAGTAGGGAACAC	118
STAT1	GACCACCTCTCTTCCTGTCG	CTGCCAACTCAACACCTCTG	169
β-actin	TTCTTTGCAGCTCCTTCG	TTCTGACCCATTCCCACC	197

The primer sequences for selected genes involved in BMP3 and TGF-β1 signaling.
